# Laparoscopic removal of an ingested fish bone from the head of the pancreas: case report and review of literature

**DOI:** 10.11604/pamj.2020.36.123.23948

**Published:** 2020-06-25

**Authors:** Francesk Mulita, George Papadopoulos, Stelios Tsochatzis, Ioannis Kehagias

**Affiliations:** 1 Department of General Surgery, General University Hospital of Patras, Achaia, Greece

**Keywords:** Foreign body, pancreas, fish bone, laparoscopic surgery

## Abstract

Most ingested foreign bodies pass spontaneously through the gastrointestinal tract and only 1% of them can perforate or penetrate the wall of stomach and duodenum and migrate into organs, such as the liver and pancreas. We report herein the case of a 59-year-old woman who presented to the emergency department with epigastric pain and fever. Computed tomography of the abdomen revealed a linear foreign body that perforate the posterior wall of the prepyloric region of the stomach. The foreign body was removed laparoscopically in one piece and was identified as a 3-cm-long fish bone. The patient recovered without complications and was discharged on the 4th postoperative day. Pancreatic foreign body is a rare entity and laparoscopic removal is warranted in majority of cases.

## Introduction

Foreign bodies in the pancreas are rare and usually caused by sharp objects, such as fish bones, sewing needles or toothpicks [1]. These objects usually pass through the anus without any complication. In 10-20% of patients endoscopic removal is required, while in 1% of patients surgery is necessary. Fish bone is an ingested foreign body which can perforate through the wall of stomach or duodenum and migrate to other surrounding organs like the pancreas and liver [2]. Many life-threatening complications like pancreatitis, pancreatic abscess and pseudoaneurysm can be occurred, when a foreign body reach pancreas [3]. There are very few reported cases in which an ingested foreign body penetrated the gastrointestinal tract and migrated into the head of the pancreas. We herein report a case of a laparoscopic removal of an ingested fish bone that was embedded into the pancreas.

## Patient and observation

**Figure 1:**
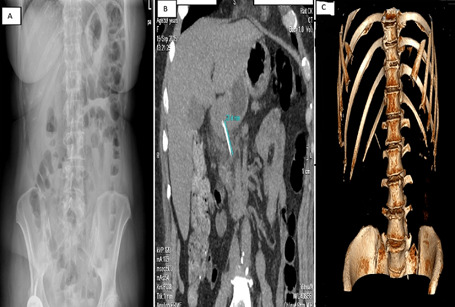
abdominal radiography showed no abnormalities (A); computed tomography (CT) scan of the abdomen revealed a linear, hyperdense, foreign body (B,C)

**Figure 2:**
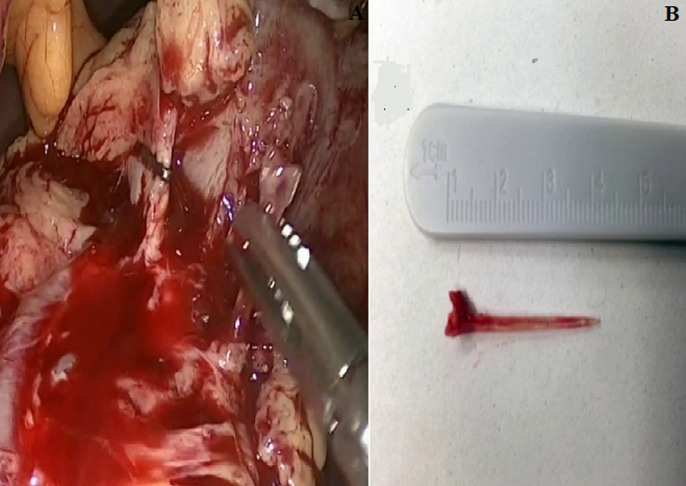
a linear foreign body was found between the prepyloric region of the stomach and the pancreatic head and was safely removed from both pancreas and stomach laparoscopically (A); the foreign body was identified as a 3-cm-long fish bone (B)

A 59-year-old female with hypertension and hyperthyroidism presented to our hospital with fever and epigastric pain for 2 days which was not associated with heartburn, vomiting, melena or haematemesis. On examination, the patient’s temperature was 37.4°C, heart rate was 97 beats per minute, blood pressure was 178/91 and respiratory rate was 19 breaths per minute. Her abdomen was soft, without distension and with no evidence of palpable mass. Her routine blood tests including haemogram, C-reactive protein level, liver and renal function test, serum amylase and lipase were normal. Chest and abdominal radiography showed no abnormalities (Figure 1A). A further computed tomography (CT) scan of the abdomen revealed a linear, hyperdense, foreign body (Figure 1B, C) that perforate the posterior wall of the prepyloric region of the stomach and there was no evidence of free air, pancreatitis or abscess formation. A further computed tomography (CT) scan of the abdomen revealed a linear, hyperdense, foreign body (Figure 1B, C) that perforate the posterior wall of the prepyloric region of the stomach and there was no evidence of free air, pancreatitis or abscess formation. An attempt was made by gastroenterologists to remove the foreign body endoscopically. This was unsuccessful because the foreign body was not detected and patient agreed to undergo a surgery. A laparoscopic surgery was performed. During the operation, the greater omentum was separated from the transverse colon and the lesser sac was opened with the help of laparoscopic instrument. A linear foreign body was found between the prepyloric region of the stomach and the pancreatic head (Figure 2A) and was safely removed from both pancreas and stomach in one piece laparoscopically. The foreign body was identified as a 3-cm-long fish bone (Figure 2B). Bleeding was controlled by pressure with a gauge and no suture repair was performed, because the penetrated gastric wall was small and no leak was observed. No drain was placed in the lesser sac. The procedure was total laparoscopic and there was no reason for conversion to an open approach. The patient did not have any postoperative complication and she was mobilized 12hours postoperatively. She was started on oral diet on postoperative day two and was discharged on postoperative day four. She is doing well for 1 year after the surgery.

## Discussion

Sharp foreign bodies like fish bone, chicken bone, tooth pick and sewing needle may be ingested accidentally or otherwise [1]. Most of them are excreted and only 1% can cause perforation. There is a high risk of perforation, when the foreign body has sharp ends at one or both ends. The usual region of perforation occurs at points of narrowing in the GI tract. Many case reports are of penetration into the pancreas, which suggests that the narrowing of the pylorus may be the mechanism by which foreign objects penetrate into the pancreas [2]. Fish bones are one of the most common ingested foreign bodies. A recent review of the English literature revealed only seven cases of an ingested fish bone that penetrated through the gastrointestinal tract and migrated into the pancreas [4-10], as demonstrated in Table 1. In these cases, a fish bone penetrated the stomach [4,5,8,10] or the duodenum [6,7,9]. Open surgical approach was preferred in the 6 of them and only in one case the ingested fish bone was removed laparoscopically with great success [10]. CT scan is useful for detecting foreign bodies like an ingested fish bone. It usually reveals a linear, hyperdense, foreign body corresponding to a bone [10]. On the basic of findings on CT, treatment of choice for penetration of the gastrointestinal tract by an ingested fish bone consists of endoscopic removal, surgical intervention, abscess drainage if necessary, and administration of appropriate antibiotics [10]. Early diagnosis and prompt treatment are mandatory to improve the prognosis of this rare condition. A mortality rate of 10% has been reported because of missed or delayed diagnosis [8]. Since many foreign bodies migrate to the pancreas, a laparoscopic approach may be beneficial over open procedures because it allows the surgeon to approach the lesser sac with minimal manipulation of surrounding tissues while being aided by optimal magnification and illumination [2]. For diagnosed abdominal foreign body extraction, laparoscopic approach should be preferred especially in stable nonacute patients, because of its advantages of less postoperative pain, lower incidence of wound infection, and minimal surgical stress [3]. In the present case, we first attempted to remove the foreign body endoscopically, but failed because it was not detected. Our patient underwent a laparoscopic removal of an ingested fish bone and recovered without complications.

**Table 1:** cases of an ingested fish bone that penetrated through the gastrointestinal tract and migrated into the pancreas, classified by location and the surgical approach

Author	Year	Location	Surgery
Goh BK	2004	Stomach	Open
Wang WL	2008	Stomach	Open
Yasuda T	2010	Duodenum	Open
Symeonidis D	2012	Duodenum	Open
Huang YH	2013	Stomach	Open
Gharib SD	2015	Duodenum	Open
Mima K	2018	Stomach	Laparoscopic

## Conclusion

Cases with foreign bodies in the pancreas are very rare and usually caused by sharp objects like an ingested fish bone. The laparoscopic approach before open surgery could be performed safely for the removal of an ingested fish bone embedded in the pancreas. Laparoscopic minimally invasive surgery should be preferred to open surgery due to its advantages.
